# Toll-like receptors as a missing link in Notch signaling cascade during neurodevelopment

**DOI:** 10.3389/fnmol.2024.1465023

**Published:** 2024-11-27

**Authors:** Mario Stojanovic, Svjetlana Kalanj-Bognar

**Affiliations:** ^1^Laboratory for Neurochemistry and Molecular Neurobiology, Croatian Institute for Brain Research, School of Medicine, University of Zagreb, Zagreb, Croatia; ^2^Laboratory for Cell Biology and Signalling, Department for Molecular Biology, Institute Ruđer Bošković, Zagreb, Croatia; ^3^Department for Chemistry and Biochemistry, School of Medicine, University of Zagreb, Zagreb, Croatia

**Keywords:** neurodevelopment, neural progenitor cells, signaling pathways, cell–cell communication, behavior pattern

## Abstract

Neurodevelopment encompasses a complex series of molecular events occuring at defined time points distinguishable by the specific genetic readout and active protein machinery. Due to immense intricacy of intertwined molecular pathways, extracting and describing all the components of a single pathway is a demanding task. In other words, there is always a risk of leaving potential transient molecular partners unnoticed while investigating signaling cascades with core functions—and the very neglected ones could be the turning point in understanding the context and regulation of the signaling events. For example, signaling pathways of Notch and Toll-like receptors (TLRs) have been so far unrelated in the vast body of knowledge about neurodevelopment, however evidence from available literature points to their remarkable overlap in influence on identical molecular processes and reveals their potential functional links. Based on data demonstrating Notch and TLR structural engagement and functions during neurodevelopment, along with our description of novel molecular binding models, here we hypothesize that TLR proteins act as likely crucial components in the Notch signaling cascade. We advocate for the hypothesized role of TLRs in Notch signaling by: elaborating components and features of their pathways; reviewing their effects on fates of neural progenitor cells during neurodevelopment; proposing molecular and functional aspects of the hypothesis, along with venues for testing it. Finally, we discuss substantial indications of environmental influence on the proposed Notch-TLR system and its impact on neurodevelopmental outcomes.

## 1 Notch and Toll—structural and signaling features

**Figure 1:**
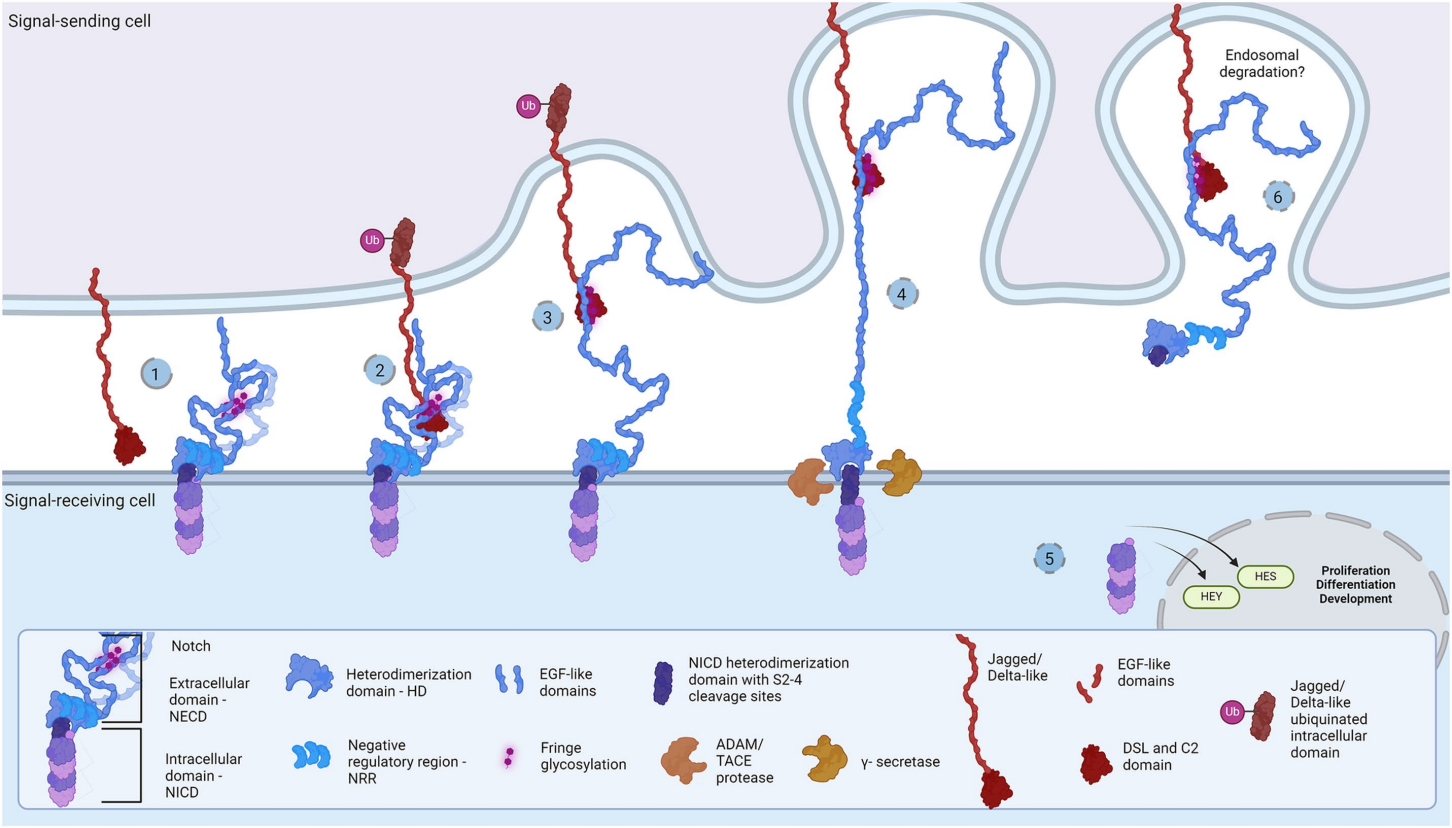
Representation of the Notch signaling pathway: The core proteins Notch and DSL ligands, Jagged and Delta-like, bind in *cis* (inhibition) and *trans* (activation) modes. Notch is a dimer of intracellular (Notch intracellular domain, NICD) and extracellular domain (Notch extracellular domain, NECD). (1) The protective dome-like NECD is glycosylated by Fringe glycotransferases. DSL ligand is composed of EGF-like domains with a Notch-interacting DSL/C2 domain. Posttranslational modifications regulate ligand affinity. Upon binding (2), the interaction groove firm binding and tension induction triggers endocytosis of DSL ligand (3) and NECD dissociation from NICD. Exposed NCID is cleaved by ADAM and γ-secretase (4) to release transcription activator NICD domain that regulates HES and HEY gene expression (5). The NECD/DSL complex undergoes endocytosis and is proposed to be degraded in the endosome (6). Created in BioRender. BioRender.com/f38j163.

During neurodevelopment, neural progenitor cells (NPCs) settled in the neuroectodermal zone act as a self-sustainable pool of cells that subsequently differentiate into neurons, oligodendrocytes, and astrocytes. In the decision-making time points, NPCs mobilize crucial developmental pathways, creating the molecular crossroads for diverse cellular fates. Across metazoan clades, the Notch signaling cascade propels developmental mechanisms and represents a fundamental pathway for development of the central nervous system (CNS) (Gaiano and Fishell, 2002; Stasiulewicz et al., 2015; Gozlan and Sprinzak, 2023). Architecture and molecular organization of the developing brain is under the influence of Notch in synergy with multiple pathways, such as Sonic Hedgehog (Shh) and Wnt signaling pathways (Akai et al., 2005; Keilani and Sugaya, 2008; Jacobs and Huang, 2019, 2021; Marczenke et al., 2021). Being a cell contact-dependent pathway, the Notch signaling hub events occur on the membranes of signal-sending and signal-receiving cells (Fiúza and Arias, 2007; D’Souza et al., 2010; Lovendahl et al., 2018). The principle of the signaling cascade comprises binding of Notch receptors on the signal-receiving cell with the Delta/Serrate/LAG-1(DSL) family of ligands, mostly Jagged and Delta-like (J/D), on the signal-sending cell. The juxtacrine J/D complex association and endocytosis-induced tension generation are vital for exposure of S2 and S3 cleavage sites (Sprinzak and Blacklow, 2021). A series of ADAM and γ-secretase proteolytic cleavages release the membrane-bound Notch intracellular domain (NICD)(Brou et al., 2000; Mumm et al., 2000; Sanchez-Irizarry et al., 2004; Fiúza and Arias, 2007; Lovendahl et al., 2018; Sprinzak and Blacklow, 2021; Wolfe and Miao, 2022), and its nuclear translocation activates Notch-targeted HES and HEY genes (Figure 1) (Kageyama and Ohtsuka, 1999). The selection of receptor and ligand isoforms sets up a large number of binding combinations related to unique (Six et al., 2003; Gordon et al., 2007; D’Souza et al., 2010; Lovendahl et al., 2018; Zeronian et al., 2021; Gozlan and Sprinzak, 2023) molecular and cellular outcomes. Let us first describe Notch and its J/D ligand extracellular engagement mechanism in order to explain in more depth the hypothesis about Toll-like receptors (TLRs) as novel and essential partners in Notch signaling during neurodevelopment.

Notch receptors (Notch1-4) are transmembrane proteins comprised of two non-covalently bound heterodimers (Sanchez-Irizarry et al., 2004; Lovendahl et al., 2018). The extracellular monomer (Notch extracellular domain, NECD) consists of multiple epidermal growth factor (EGF) domains with DSL-interacting EGF domains wrapping the mechanosensory Negative Regulatory Region (NRR) domain (Sanchez-Irizarry et al., 2004; Lovendahl et al., 2018; Sprinzak and Blacklow, 2021). The dimerization complex resembles a mushroom: buried under folds of the LIN-12/Notch repeat (LNR) domain, the heterodimerization (HD) domain protects the intracellular monomer protruding stem with S2 and S3 cleavage sites (Lovendahl et al., 2018; Sprinzak and Blacklow, 2021). In mammals, two Jagged (Jag1-2) and two out of three Delta-like (Dll1, Dll4) ligands bind Notch and pull off the protecting NECD to reveal the cleavage sites, while Dll3 does not bind Notch and is localized in endosome (Nichols et al., 2007; Musse et al., 2012; Lovendahl et al., 2018; Sprinzak and Blacklow, 2021). The JD’s extracellular portion also has multiple EGF repeats between the N-terminal C2-like immunoglobulin domain connected to the DSL domain and the cysteine-rich region proximally to the membrane (Lovendahl et al., 2018). Both proteins form rod-like open solenoids as a result of variable EGF repeats (Bork et al., 1996; Wouters et al., 2005; Kelly et al., 2010) and are glycosylated which is a standard structural feature of adhesion molecules (Moloney et al., 2000; Chen et al., 2001; Wouters et al., 2005; Yu et al., 2021). The extracellular portion of the Notch receptor responsible for J/D engagement is between EGF8 and EGF13, while the adhering part of the J/D are C2-like and DSL domains (Cordle et al., 2008a; Musse et al., 2012; Luca et al., 2015; Handford et al., 2018). C2-like domain has lipid-binding properties probably acting as a membrane proximity sensor: the lipids in question are glycosphingolipids (GSLs) in *Drosophila* (Hamel et al., 2010) and phospholipids (PL) in mammals (Ca^2+^-dependent in Jag) (Chillakuri et al., 2013; Suckling et al., 2017; Handford et al., 2018). Notch EGF10-12 and Jag/Dll C2-DSL interaction groove covers an area of approximately 1,100 Å^2^ and provides variety and strength of interactions (Cordle et al., 2008a, 2008b; Luca et al., 2015; Handford et al., 2018), i.e., stabilization of glycosylated residues, hydrophobic and H-bond establishment, salt-bridge formation, and Ca^2+^-dependent structural rigidity induction, enable the conformational change, generate tension which activates endocytosis and NECD dissociation (Nichols et al., 2007; D’Souza et al., 2010; Musse et al., 2012). This action reveals NICD cleavage stem while following proteolytic action leads to subsequent intracellular signaling events (Nichols et al., 2007; Musse et al., 2012; Luca et al., 2017; Sprinzak and Blacklow, 2021). The destiny of the Notch/J/D ligand complex after endocytosis is elusive but is believed to undergo endosomal degradation. In order to bind the Notch receptor, J/D has to be in ‘flexed’ conformation which exposes the binding groove, and it is supposed that J/D spontaneously transitions between ‘flexed’ and ‘stiff’ conformation (Luca et al., 2015, 2017; Handford et al., 2018). The dynamics and steps of conformational transition, how exactly the Jag and Dll achieve their flexed conformation, find and bind Notch, and what happens with the pulled NECD/J/D ligand complex in the signal-sending cell remains unclear. This leaves space for speculations: is there an additional molecule which delivers and presents ligands to Notch receptors; does the signal-sending cell recognizes the final response and if so is there a response-reading machinery in the signal-sending cell? Here we aim to clear up these questions and offer a novel perspective on the Notch signaling cascade, by introducing the TLR family as potential member of the Notch cascade.

The Toll-like receptor (TLR) family of proteins first emerged in Cnidaria (Beutler and Rehli, 2002; Brennan and Gilmore, 2018; Liu et al., 2019) and has since evolved as a patterning tool in development (Barak et al., 2014; Noordstra and Yap, 2021; Umetsu, 2022; Weiss and O’Neill, 2022; Sommariva et al., 2023) and a pattern-recognition tool in immunity (Crack and Bray, 2007; Ma et al., 2007; Park et al., 2009; Scheffel et al., 2012; Anthoney et al., 2018; Chen et al., 2019). The first mention of TLR proteins was the Toll receptor in *Drosophila melanogaster* embryology (Parthier et al., 2014; Al Asafen et al., 2020; Umetsu, 2022), where it serves as a molecular agent for cellular organization in dorsoventral body symmetry formation. Namely, the dosage of ligand Spätzle is measured with the Toll receptor and translated into Dorsal, Snail, and Twist protein gradients, resulting in a cellular patterning of ectoderm, neurogenic ectoderm, and mesoderm (Umetsu, 2022). Later, the same receptor was revealed as a signaling hub in innate immunity upon external pathogen stimuli (Takeda and Akira, 2004; Botos et al., 2011). These two functions are preserved with evolutionary modifications in mammals. For instance, mammalian TLR actions associated with innate immunity differ from *Drosophila’*s due to an additional direct ligand-binding capacity; TLR expression in NPCs and involvement in developmental processes, without apparent endogenous ligand, confirms its role in neurodevelopment. With that said, let us explore the structural and functional features of TLRs that will speak in favor of the hypothesis.

**Figure 2:**
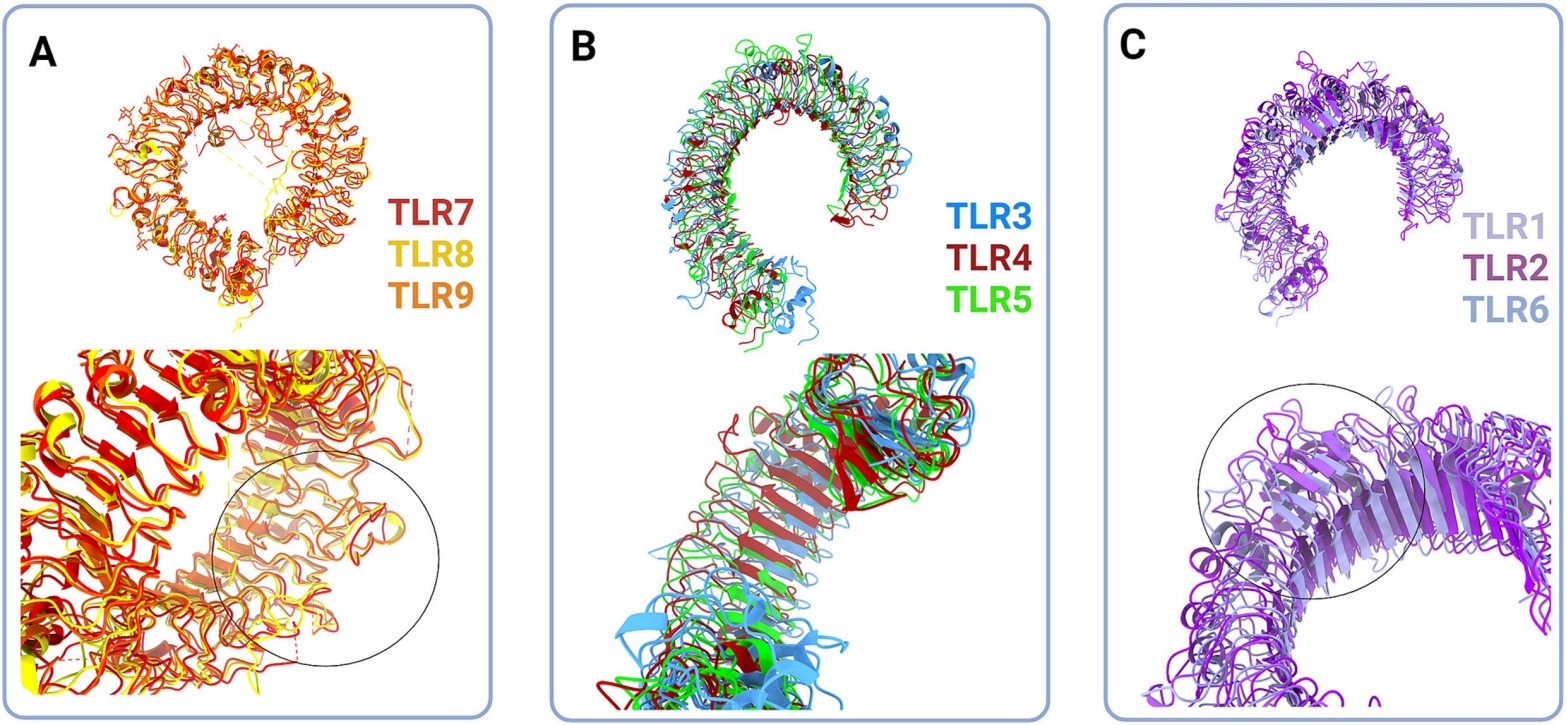
Structural classes of Toll-like receptors (TLRs) are long (A and B) and short (C) TLRs. (A) Superimposed structural models of TLR7 (red, hTLR7 PDB:7CYN), TLR8 (yellow, hTLR8 PDB:7R54) and TLR9 (tangerine, mTLR PDB:3WPG) (upper panel). Zooming in on the loops on dimerization side of long TLRs reveals that loops enclose cavity between two dimers (bottom panel, circle). (B) Superimposed structural models of TLR3 (blue, hTLR3 PDB:7C76), TLR 4 (deep red, hTLR4 PBD:3FXI) and TLR5 (green, hTLR5 PDB:3J0A) (upper panel). Zoom in on the LRR types in TLR3, TLR4 and TLR5 (bottom panel). (C) Superimposed structural models of TLR1 (lilac, hTLR1 PDB:6NIH), TLR2 (purple, hTLR2 PDB:6NIG), and TLR6 (light blue, mTLR6 PDB: 3479) (upper panel). Closing up of TLR2, TLR1 and TLR6 β sheets on dimerisation side dictate the ligand cavity depth on top of TLR monomer (bottom panel). Created in BioRender. BioRender.com/o67p300.

**Figure 3:**
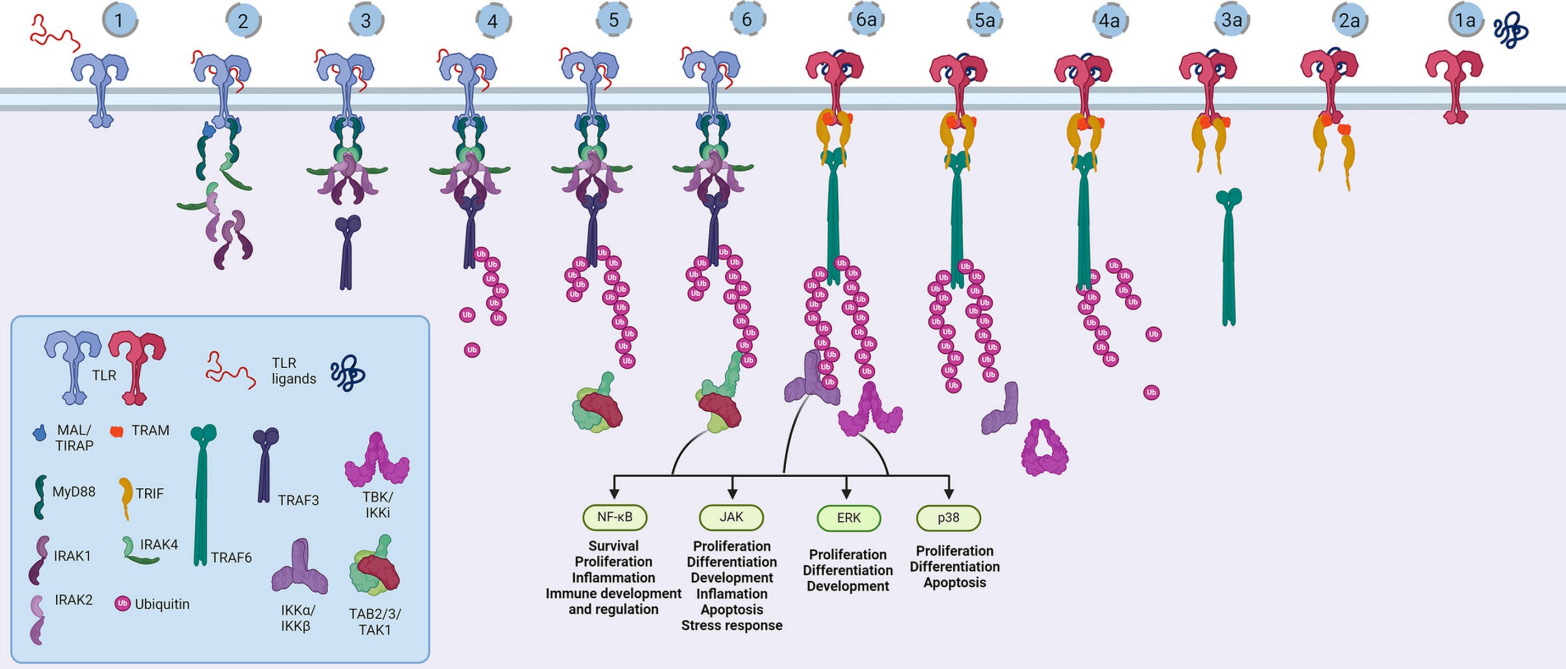
Representation of the Toll-like receptor pathway. Toll receptors scaffold signalosomes in membrane proximity, e.g., TLR dimers scaffold Mydosome (TLR2/1, TLR2/6, TLR4, TLR5, TLR7, TLR8 and TLR9) (1–6) and TRIFosome (TLR3 and TLR4) (1a-6a). Ligand binding to TLR (1) primes Mydosome. TLR recruits MAL/TIRAP,MyD88, IRAK1/2 and IRAK4 scaffold in hexameric structures (2) activating TRAF3 and TRAF6 (3) which are able to auto-ubiquinate (4). Poly-ubiquitin chains associate (5) and activate TAB2/3/TAK1 kinases (6). Trifosome is primed with TLR ligand binding (1a). Associated TRAM and TRIF (2a) activate TRAF3 directly (3a) and TRAF6 in a RIP1-dependant fashion. TRAF3 polyubiquitination (4a) recruits (5a) and activates TBK/IKKi kinases (6a). These kinases regulate IKKα/IKKβ kinases and input of NF-κB, JAK, ERK and p38 regulated genes and proceses. Created in BioRender. BioRender.com/a29t370.

**Figure 4:**
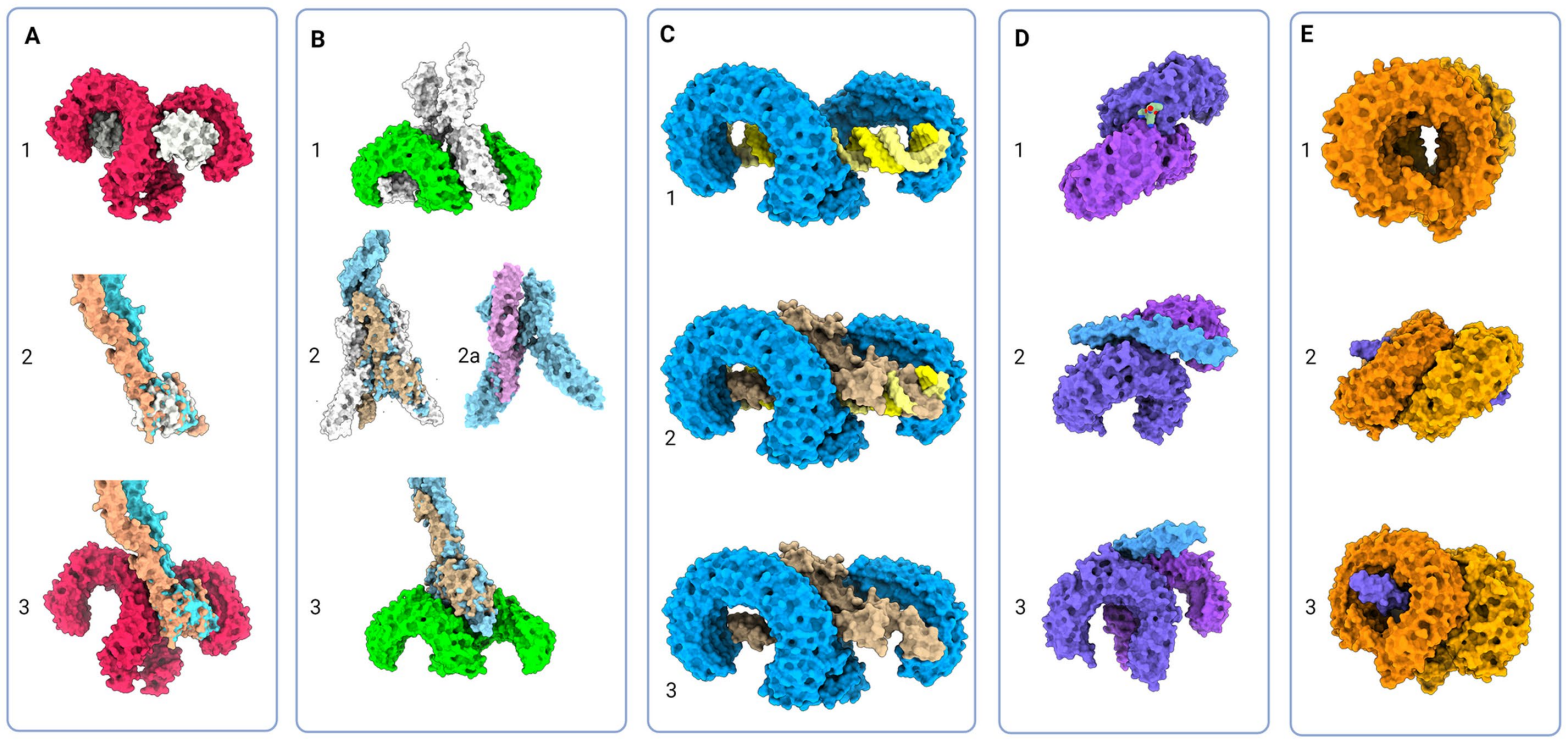
Protein models of TLRs with natural ligands and superimposition of TLR and Notch signaling components; (A) 1 - Molecular visualization of TLR4 dimer with coreceptor MD-2 (PDB:3FXI), 2 – Aligned models of MD-2, Jagged (apricot, PDB:5UK5) and Delta-like (tan, PDB:4XL1), 3 – Proposed model of Jagged and Delta-like in complex with TLR4; (B) 1 – TLR5 model in complex with flagellin (PDB:3 V47), 2 – Flagellin model aligned with NECD/Delta-like complex (tan, PDB:4XLW, light blue, PDB:4XL1), 2a – Flagellin model (PDB:3 V47) aligned with APP core domain (lilac, PDB:3NYL), 3 – Proposed model of NECD/Delta-like in complex with TLR5; (C): 1 - TLR3 in complex with dsDNA (PDB:7DAS), 2 – Superimposed NECD/Delta-like complex over dsRNA in complex with TLR3, and 3 – Theoretical binding model of TLR3 and NECD/Delta-like complex; (D) 1 - TLR1/2 in complex with LPS (PDB:2Z7X), 2 and 3 - Theoretical model of TLR2/1 interaction with Notch EGF-like domains (PDB:4D0E) (E) 1 - Model of TLR7 dimers (PDB:5GMF), 2 and 3 – Theoretical model of EGF-like domains (PDB:4D0E) in complex with TLR7. Created in BioRender. BioRender.com/t26j594.

Structurally, the Toll-like receptors form an open solenoid by packing leucine-rich repeats (LRRs) in a horseshoe-shaped extracellular domain with adhesive and binding capacity (Jin et al., 2007; Kang et al., 2009; Park et al., 2009; Botos et al., 2011; Salunke et al., 2012). The function of LRR proteins depends on their structure (specifically, radii and number of LRR modules) (Kobe and Deisenhofer, 1994; Kobe and Kajava, 2001; Enkhbayar et al., 2004) and regarding the LRR composition, TLRs are divided into long and short structures (Figure 2). The intracellular domain of TLRs is a Toll-interleukine-1 receptor (TIR) homology domain that targets TIR-containing proteins, e.g., MyD88 and TRIF, activating TAB2/3/TAK1 and TBK1 relay, TRAF3/6-operated molecular switch. In brief, TLR1,2,4–9, dimers bind TIRAP and MyD88 that scaffold IRAK4/IRAK1/2 and activated TRAF3/6 proteins. Described down-stream effects of signalization upon TLR3 and TLR4 dimerization (Beutler and Rehli, 2002; Ge et al., 2002; Matsumura et al., 2003; Verstak et al., 2009; Smoak et al., 2010; Tukhvatulin et al., 2010) which inlude TRAM and TRIF adaptors, activation of TRAF3 and TRAF6, formation of TAB2/3/TAK1 signalosome and regulation of IKK and MAPK signaling events are presented in Figure 3. TRAF3 activates additional families of IKK-like kinases (TBK1, IKKi). Activated genetic readouts and protein machinery are described in immune answer (Vabulas et al., 2001; Jack et al., 2019; Chen et al., 2021), proliferation and differentiation (Lathia et al., 2008; Ulrich et al., 2015; Song et al., 2019; Mann et al., 2023), mature and apoptotic phenotypes (Cameron et al., 2007; Ma et al., 2007; Stridh et al., 2011; Ishizuka et al., 2013; Li et al., 2013, 2020; Shi et al., 2013; Church et al., 2016; Abdul et al., 2019; Werkman et al., 2020). The library of TLR-activating ligands induces TLR homodimerization in antiparallel orientation, except for versatile TLR2, which builds heterodimers with TLR1, TLR4, or TLR6 in a ligand-dependent fashion (Kang et al., 2009; Schenk et al., 2009; Fernandez-Lizarbe et al., 2013; van Bergenhenegouwen et al., 2013; Koymans et al., 2015; Liu et al., 2019; Ved et al., 2021; Galleguillos et al., 2022). TLR structural features, LRR concave β-sheet, and outer α-helices surface delineate their specificity to ligands, creating a multifaceted recognition hub (Figures 2, 4A) (Werling et al., 2009). Membrane localization of TLR2, TLR4, and TLR5 enables them to recognize and bind di-and triacylated lipoproteins (TLR2/1, TLR2/6) (Jin et al., 2007; Kang et al., 2009), LPS presented by coreceptors (TLR4) (Kim et al., 2007; Park et al., 2009) and flagellin (TLR5) (Smith and Ozinsky, 2002; Yoon et al., 2012). Endosomal TLRs bind viral dsRNA (TLR3), ssRNA (TLR7, TLR8) and ssDNA (TLR9) (Choe et al., 2005; Wang et al., 2006; Botos et al., 2009; Gosu et al., 2019). Mammalian TLRs are designed to fit and bind pathogen-associated molecular pattern molecules (PAMPs), damage-associated molecular pattern molecules (DAMPs), and protein coreceptors (Akashi-Takamura and Miyake, 2008; Park and Lee, 2013; van Bergenhenegouwen et al., 2013). Interestingly, the evolutionary older ligands, particularly in *Drosophila* development, are protein ones, and we could consider that TLRs’ protein-binding mode of action makes a conserved *modus operandi* in developmental processes. TLRs, specifically TLR4, recognize and interact with distinct structural features of protein ligands (Vabulas et al., 2001; Kim et al., 2005; Liu et al., 2012; van Bergenhenegouwen et al., 2013; Mehmeti et al., 2019; Ved et al., 2021), particularly protein folds and carbohydrate moieties, opening the possibility that a (glyco)protein could be a potential TLR4 neurodevelopmental ligand. The protein coreceptors that bind to mammalian TLR4 have a distinct C2 globulin fold with a lipid binding pocket; e.g., globulin MD-2 in a complex with LPS is TLR4 binding partner (Gioannini et al., 2004; Kim et al., 2007; Schumann, 2011). TLR4/MD-2 quaternary complex consists of two MD-2 molecules engaged with both TLR4 monomers. Unique structural features of TLR4 allow MD-2 binding at the concave dimerization face of the first and the back of the second TLR4 monomer (Figure 4A) (Kim et al., 2007). Protein MD-2 and Jag/Dll C2-like domains have evolutionarily conserved folds, showing overall similarity between overlapped models of MD-2 and Jag/Dll C2-like domains (Figure 4A2). This structural similarity between the Notch ligands and the TLR4 coreceptor is the first indication that TLRs possibly act as a binding partners of Notch ligands.

## 2 Notch and Tolls in neurodevelopment

To investigate the hypothesis on shared Notch and Toll signaling mechanisms with implications for developing nervous system, we demonstrate systematically the striking functional overlaps of Notch and TLR signaling during neurodevelopment documented in the literature (Table 1).

**Table 1:** Cell-specific expression and functions of Notch and TLR signaling pathways during neurodevelopment.

Cellular type	Notch pathway	TLR pathway
Neural progenitor cells (NPCs)	Radial glia differentiation is induced by Notch1 (Patten et al., 2003, 2006) Notch signaling regulates proliferation of NPCs (Kageyama et al., 2009) Olfactory epithelium stem and progenitor cells utilize Notch (Schwob et al., 2017) Notch as NPC commitment switch (Zhou et al., 2010) Overlapping and differential expression of Notch1-3, Jag1-2, Dll1 and Dll3 (Irvin et al., 2001, 2004) Differential expression of Notch1, Jag1, Jag2, Dll1, Dll3, Hes 1,3,5, Numb and Numblike (Stump et al., 2002); lack of Dll1 leads to impaired neurodevelopment (Arzate et al., 2022) Brain lipid-binding protein as mediator of Notch signaling in radial glial cells (Anthony et al., 2005)	NPCs proliferation is regulated by TLR3 (Lathia et al., 2008; Yaddanapudi et al., 2011) NPCs proliferation is regulated by TLR2 (Okun et al., 2010) Maternal exposure to TLR3 ligands promotes proliferation of NPCs (Baines et al., 2020) Maternal exposure to TLR2, TLR4 ligands enhances NPC proliferation and expands neocortex (Mann et al., 2023) TLR2 and TLR4 differentially regulate spinal cord NPCs (Sanchez-Petidier et al., 2022) Retinal PC proliferation is under TLR4 control (Shechter et al., 2008) Differential expression of TLRs in mammalian and *Drosophila* brain (Shmueli et al., 2018) Expression of TLRs in human CNS (Bsibsi et al., 2002) Adult hippocampal neurogenesis is under TLR control (Rolls et al., 2007)
Neuron	Dll1 induces NPC commitment to neuronal phenotype (Kawaguchi et al., 2008) Dll3 overexpression and translocation to the membrane, as Dll1, promotes and maintains neurogenesis (Ladi et al., 2005) Jag1 influences neuronal division and periglomerular interneurons (Blackwood et al., 2020) Dll1 intrecellular domain regulates dorsal root ganglion development (Okubo et al., 2021) Jag2 in motor neuron generation (Rabadán et al., 2012) Notch signaling influences dopaminergic neuron number (Trujillo-Paredes et al., 2016). Notch and Dll guide axon pathfinding (Giniger et al., 1993) Notch1 and Dll1 regulate mamalian neurite development (Franklin et al., 1999) Glia and neuron Notch crosstalk in establishment of longitudinal axon connections (Kuzina et al., 2011) Notch signaling-mediated axon outgrowth (Shi et al., 2011) Notch regulates neurite outgrowth by contact-inhibition (Šestan et al., 1999) Notch regulates neuronal cell faith and dendrite morphology (Breunig et al., 2007)	TLRs regulate differentiation of NSCs into neurons (Ulrich et al., 2015) After brain injury, TLR2 promotes neurogenesis (Seong et al., 2018) TLR4 ligands have anti-neurogenic effect (Zaben et al., 2021) TLR4 has a role in amygdala GABAergic transmission (Varodayan et al., 2018) TLR3 has potential negative role in axon growth (Cameron et al., 2007) TLR8 role in developing neurons and axons (Ma et al., 2007) TLR3 contributes to Wallerian degeneration of white matter (Lee et al., 2017) TLRs in neuronal morphogenesis (Liu et al., 2014) TLRs regulate neurogenesis and synaptic physiology (Chen et al., 2019)
Astrocyte	Jag and Notch mediate retinal gliogenesis (Jin et al., 2023) Dll1 regulates Bergman glia monolayer formation (Hiraoka et al., 2013) Neurons induce NPCs astrocyte commitment through Notch signaling (Namihira et al., 2009)	Adult hippocampal NPCs differentiate into astrocytes at the expense of neurons (Rolls et al., 2007) Astrocyte populations differently respond to TLR4 and TLR3 ligands in myelination events (Werkman et al., 2020) TLRs are expressed in astroglia (Li et al., 2021)
Oligodendrocyte	Radial glia specification into oligodendrocytes is governed by Notch (Kim et al., 2008) Jag2 regulates spinal cord oligodendrocyte generation (Rabadán et al., 2012) Notch has dual role in neuron-to-oligodendrocyte switch (Tran et al., 2023) Notch signaling evidenced in oligodendrocyte genesis and homeostasis (Li C. et al., 2022)	TLR2 and TLR4 differentially influence oligodendrocyte formation after inflammation (Schonberg et al., 2007) Inhibition of TLR2 stimulates OPCs and TLR3 inhibition induces apoptosis (Bsibsi et al., 2012) TLR2 tolerance enhances remyelination in CNS (Wasko et al., 2019) TLR2/4 ligand, HMBG1, reduces OPC number (Ved et al., 2021) Transplanted OPCs and inflammation response in myelination (Setzu et al., 2006) The lack of TLR4 impairs oligodendrocyte formation after injury (Church et al., 2016)

Described functions of Notch signaling during neurodevelopment encompass NPCs number, identity, spatial organization (Irvin et al., 2001, 2004; Stump et al., 2002; Patten et al., 2006; Keilani and Sugaya, 2008; Kim et al., 2008; Zhou et al., 2010; Rabadán et al., 2012; Blackwood et al., 2020; Marczenke et al., 2021; Mase et al., 2021; Okubo et al., 2021; Li C. et al., 2022; Tran et al., 2023) and connectome formation events (Franklin et al., 1999; Šestan et al., 1999; Breunig et al., 2007; Kuzina et al., 2011; Shi et al., 2011). Notch signaling seems to be crucial for asymmetric NPCs division (Casas Gimeno and Paridaen, 2022) and may be viewed as a switch between NPC differentiation, neurogenesis, and gliogenesis. Also, Notch1 is implicated in radial glia (RG) identity establishment and differentiation to ventral RG (Patten et al., 2003, 2006; Mase et al., 2021). These findings confirm that Notch orchestrates morphology, outgrowth and density ratio of neurons, oligodendrocytes, and astrocytes which eventually has implications on the axonal, dendritic, and synaptic network formation (Šestan et al., 1999; Shi et al., 2011; Kuzina et al., 2011).

Evidenced extensive impact of Notch signaling on critical neurodevelopmental events overlaps with so far known neurodevelopmental functions of TLRs. Firstly, TLR activation affects the immune response during and after (neuro)development, and its effect was observed in cooperation with the Notch pathway and as a Hh and Wnt signaling regulator, respectively (Opitz et al., 2001; Re and Strominger, 2001; Vabulas et al., 2001; Frantz et al., 2001; Matsumura et al., 2003; Leemans et al., 2005; Crack and Bray, 2007; Ziegler et al., 2007; Palaga et al., 2008; Park et al., 2008; Hu et al., 2008; Nichols et al., 2009; Lalancette-Hbert et al., 2009; Cameron and Landreth, 2010; Foldi et al., 2010; Tsao et al., 2011; Liu et al., 2012; Winters et al., 2013; Kim et al., 2013; Okun et al., 2014; Hamidi et al., 2014; Rangasamy et al., 2018; Mehmeti et al., 2019; Martínez-García et al., 2020). Secondly, the expression of TLRs on all cell types across the developing and adult brain unquestionably affirms their impact on neurodevelopment (Barak et al., 2014; Okun et al., 2019).

Clearly, a large body of evidence confirms that certain TLR functions match the scope of Notch functions during embryonic and postnatal brain development (Table 1). Observed functional overlaps indicate that different TLR and Notch proteins govern switches between cellular commitments. For example, the actions of Dll1, TLR2, and TLR4 overlap to some extent: Dll1 induces neurogenesis; loss of Dll1 and TLR4 induces proliferation and gliogenesis; the loss of TLR2 induces proliferation and neurogenesis. From that perspective, TLR2 and Dll are required for proliferation cessation, while TLR4 and Dll1 combinations become inductors of the same cellular fate, suggesting they are a part of the same signaling machinery. It has to be noted that interplay of Notch and TLR signaling has been described in parts, however majority of studies focuses on coordinated responses of the immune system components, based on widely recognized functions of TLRs in innate immunity (Hu et al., 2008; Palaga et al., 2008; Tsao et al., 2011; Shang et al., 2016). The most striking interplay between TLR and Notch in immunity was documented in the study by Hu et al. which confirmed synergistic cooperation between the Notch and TLR pathways mediated by RBP-J. Specifically, the acute TLR induction has been shown to activate HES1 and HEY1 genes, while TLR interleukin genes expression were dependant on NICD nuclear translocation and RBP-J binding. This finding fully supports here presented theory and it would be certainly interesting to confirm the same interplay during neurodevelopment. So far, direct involvement of TLRs in neurodevelopment has been underappreciated, but the herein reviewed findings indicate that TLRs stand at the core program of the nervous system development, and deciphering the role of TLR signaling, particularly as the part of the proposed TLR-Notch complex could be valuable for understanding the spatiotemporal cellular organization of CNS.

## 3 Hypothesis

We suggest that TLRs are an integral part of the Notch signaling cascade acting as a Notch signaling transductor scaffold and deciphering machinery in both signal-sending and signal-receiving cells. Events that presumably lead to Notch binding and signaling on the signal-receiving cell membrane are organized with TLR1, TLR2, TLR4, and TLR6, while TLR5, TLR3, TLR7, TLR8, and TLR9 are an answer interpreting machinery for the signal-sending cell. The proposed functions for each TLR would occur sequentially as follows: TLR4—ligand bait; TLR1,2,6 heterodimers—Notch sorter and selector; TLR5—answer catcher; TLR3—answer reader; TLR7,8 and 9—answer reader and tuner.

## 4 Proposed experimental paradigms

To prove the hypothesis, one would need to check for biochemical and physiological evidence of TLR involvement in the Notch pathway and correlate them to the functional and eventually behavior patterns. The biochemistry of the proposed system should first describe chemico-physical interactions, followed by observations using models *in vitro* and *in vivo*, and it could be investigated in several ways:

1. Interactive visualization and analysis of molecular structures utilizing available models from PDB library,^1^ bioinformatics tools such as PDBeFold^2^ and Chimera program^3^;
2. Molecular simulation, the fast-science approach using already existing structural data collected on Notch, Jag, Dll, and TLR which could be uploaded into programs providing us with information on the proposed interaction parameters (orientation, energies, contacts, electrostatic, polar, and non-polar interactions) and enabling understanding and locating target protein interactions;
3. Cross-linking mass spectrometry (XL-MS) analysis of NPCs, *in vitro* and *in vivo*, would provide a series of data regarding the molecular system of interest. Data collected in controlled conditions, the basis for the interaction matrix, would be challenged with the data collected on TLR knock-out (KO) systems and TLR-challenged systems to untangle the communication code of Notch/TLR signaling. The TLR-challenged system could also be a simulation model to investigate the dose-dependent changes introduced by the environmental setup;
4. State-of-the-art cryo-electron microscopy (EM) technology and its further advancements could be implemented for structural and topographical research of the Notch/TLR pathway. *In vitro* models of NPCs, organoids, and *ex vivo* brain imaging would provide starting material for investigation and help describe the exact 3D model and mechanics of Notch/TLR initial signaling events.

The proposed solutions for checking the hypothesis are methodologically feasible and could provide the scientific community with valuable answers. Another way to test the hypothesis would be to use data collected on individuals exposed to TLR agonists *in utero* during critical neurodevelopment stages. Such data may reveal whether TLR challenge acts on neurodevelopment and whether discrete changes in neuronal number, branching and networks strength establishes neurophenotype basis for different neurobehavioral patterns.

However, the limitations of proposed structural analysis approaches should not be overlooked and require to take into account that any posttranslational modification could be responsible for maximal efficiency in binding between Notch/Jag/TLR, thus not fully resolved from available data. Other challenges might arise from the following facts: extracellular proteins are the most lavishly modified proteins; native conformations and molecular dynamics in the cellular environment are entropy-rich and challenging to simulate; glycosylated residues are intrinsic to cellular communication and serve as recognition residues; sugar code enables additional structural recognition means and induces differential affinity towards interaction partners.

## 5 Supportive evidence for novel interactions within proposed TLR-Notch assembly

**Figure 5:**
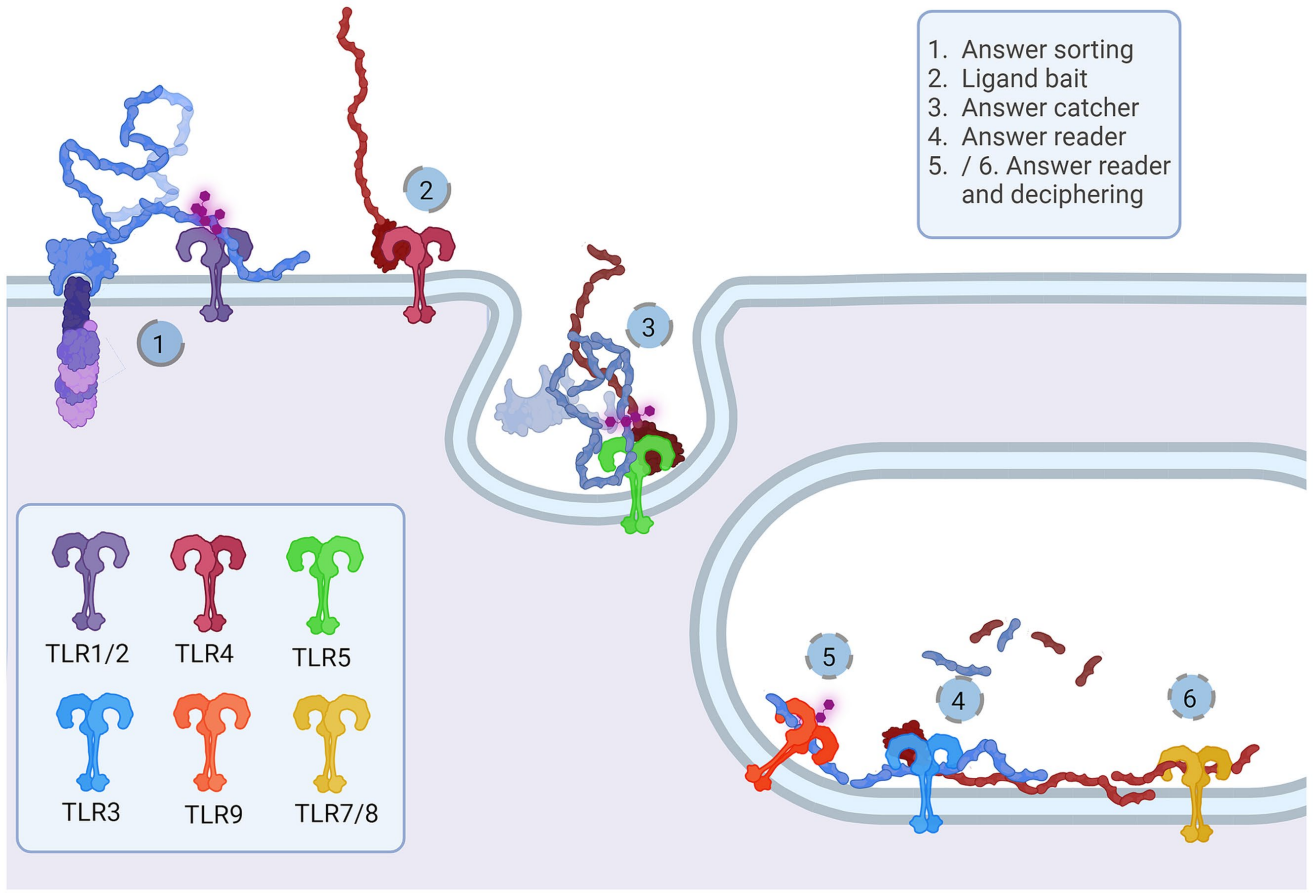
Representation of the proposed Notch-TLR pathway. Each step of the Notch cascade is accompanied by TLR dimerization and downstream signal amplification. TLR1/2/6 Notch sorting and choosing (1) binds DSL ligand fetched by TLR4 (2). The other cell senses the tension and pulls of NECD/DSL complex where TLR5 confirmes catch (5). In endosome, TLR3 signals if NECD/DSL complex is present (4) while TLR7/8/9 fine tunes the answer by examination of EGF PTMs (5 and 6). The accumulation of Notch stimulation of individual TLR proteins regulates NF-κB, JNK, ERK and p38 programes input driving cellular behavior. Created in BioRender. BioRender.com/a30x851.

Whilst there are currently no *in vivo* supportive data, the interactive visualization and analysis of molecular structures utilizing informatics tools (see footnote 2) and Chimera program (see footnote 3) yields several exciting findings. More specifically, the analyses of molecular structure and potential binding models of TLRs and Notch suggest their novel roles and interactions (also see Figures 4, 5).

The majority representation of the Notch/J/D binding model assumes Notch, Jag, and Dll as stiff sticks protruding perpendicularly to the cellular membrane and interacting similarly, deep within the extracellular matrix and away from a cell membrane. From the above-given information, Notch is a tangled dome-like protein complex that needs ligands to remove protection mechanically and enable message transduction. For this to happen, J/D must be in the precise structural orientation for groove exposure and proximity of potential binding partners (Luca et al., 2015, 2017; Handford et al., 2018). The lipophilic property of J/D C2 domain (Chillakuri et al., 2013; Suckling et al., 2017) could prime the interaction with a signal-receiving cell. It is reasonable to suggest that Jag and Dll span the distance and are loosely tensioned between two cells. Lipid-C2 interactions are an open invitation for anchoring protein that will maximize the conformational flex of the J/D structure and be a docking tool for signal-receiving cells. The cross-talk between the C2 domain of J/D and lipids on signal-receiving cells is the point where TLR4 enters the story. By interactive visualization of molecular structures we were able to observe that TLR4 recognizes J/D, binds and locks it in an active conformation, and delivers it to selected Notch (Figure 4A). The described interaction indicates a potential role of TLR4 as a molecular anchorage in signal-receiving cells. We assume that the J/D tethered with a TLR4 would provide an energetically favorable environment and support for flex conformation to ensure its availability for interaction with Notch. The TLR2 with interacting partners TLR1 and TLR6 could be a Notch sorting and ‘decision-making’ tool. Distinct TLR2 heterodimer complexes would recognize the ligand fetched by TLR4 and choose the appropriately accessorized Notch receptor. The glycosylation status of Notch receptors and J/D ligands may be a motif that allows TLR2 to sort and choose binding combinations (Figure 4D). This mode of action indicates that establishing interaction between TLR4/J/D leads to a Notch-binding tension-generating step in the cascade. We propose that TLR4 and TLR2 act as a fly ‘buttoning’ Notch and J/D. The J/D/TLR4-tether generates tension between two membranes, and the tension’s release might be a cue for the signal-sending cell membrane invagination resulting in Notch dissociation and proteolysis on the signal-receiving cell. We hypothesize that TLR4 and TLR2 support Notch and J/D ligand binding, resulting with promotion of NICD downstream cascade in signal-receiving cells.

The signal-sending cell supposedly integrates the molecular events by gathering stimuli from the pulled NECD/J/D complex. Here, based on analyzed molecular structure, we propose that TLR5 acts as an answer-receiving tool. TLR5 may sense the tension and recognize non-interacting J/D and NECDs EGF domains (Figure 4C). The NECD/J/D complex interface facing the signal-sending cell is bifurcated and shaped like ligands recognized by TLR5. With the help of TLR5, the complex is taken inside the cell, where it would be contextualized. In the endosome, degradation releases strands of Notch EGF repeats, NECD/J/D binding complex, unbound J/D ligands, and J/D ligand intracellular domain. Each degradation product is a piece of valuable information for signaling cells. We propose TLR3 recognizes and gives context to cleaved NECD/J/D binding complex (Figure 4C) while TLR7, TLR8, and TLR9 bind EGF repeats (Figure 4E) giving necessary information about cell-receiving cells responses. One single strand-recognizing TLR could also be sensing the degradation product of the cell’s unused ligands and help the signal-sending cell react appropriately to the neighboring population dynamics.

**Figure 6:**
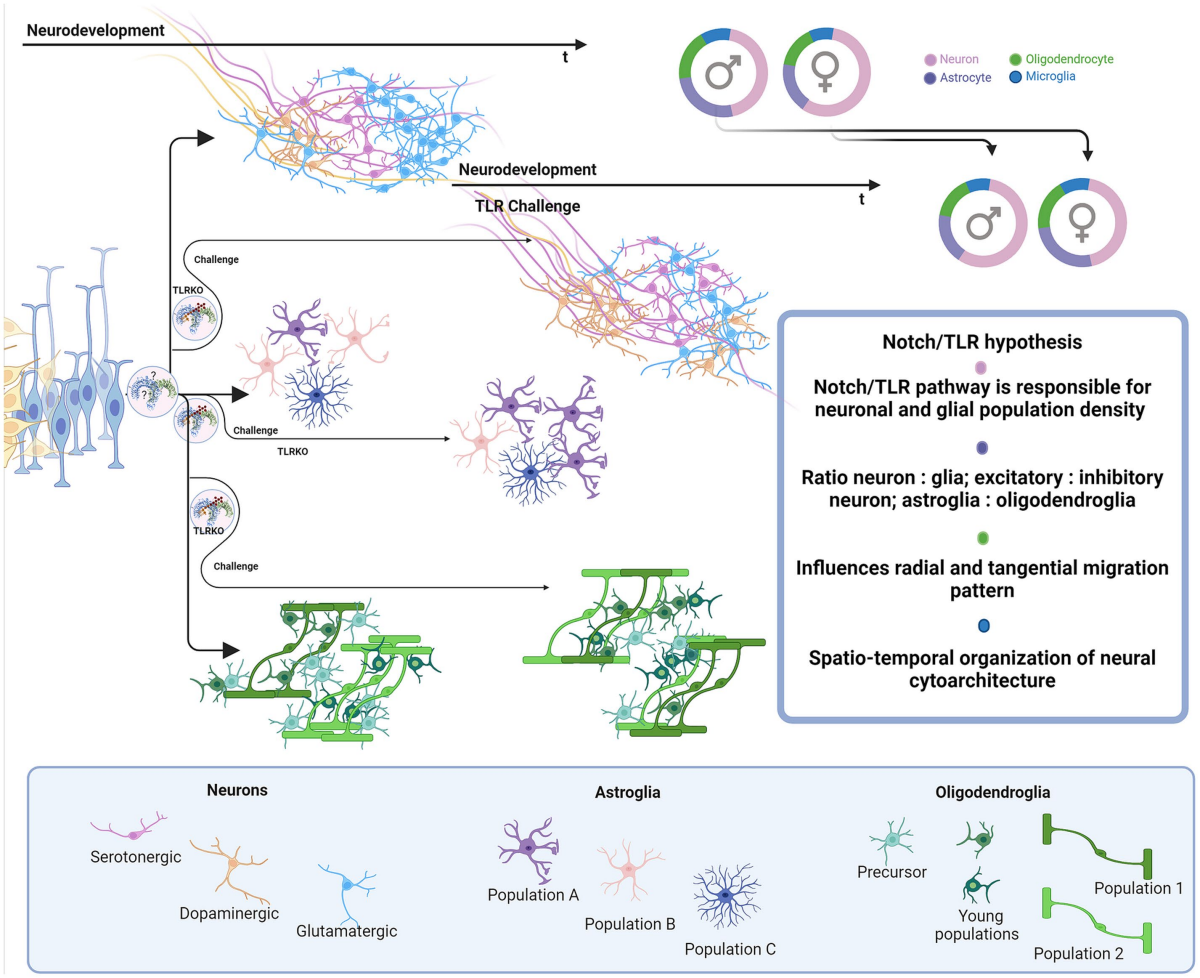
Proposed impact of Notch/TLR system on neurodevelopment. Presuming that Notch/TLR system is responsible for cellular adaptation to various stimuli and subsequent formation of cellular identity, challenged TLR system could evolve different environmentally dictated phenotypes in a sex-dependent manner. Male and female neurophenotypes could be challenged to generate variations in density and identity of cellular populations which eventually influences on neural cytoarchitecture, circuitres and behavioral phenotypes. Created in BioRender. BioRender.com/d80v300.

During differentiation, asymmetric inheritance is induced by cellular touch (Casas Gimeno and Paridaen, 2022). TLR links Notch inductors’ direct genetic input to TAB/TAK adductors’ rearrangements in cytoplasmic and membrane phenotypes to accommodate genetic identity-specific protein output. Notch signaling cascades may form an activated membrane patch that integrates changes in signal-receiving cell. Sequestering and ratio of specific IKK(α/β/γ) and IKK-like (TBK1 and IKKi) pathways are most likely needed for modulation of NF-κB-dependent and independent pathways, creating identity commitment signaling and metabolic landscapes. It is likely that TLR signaling modulates NF-κB signals induced by growth factors and synergistically affects PI3K signaling. TLR also activates IKK complex and its downstream mTORC1 and mTORC2 metabolic programmes that are highly active in neurodevelopment (Switon et al., 2017). The mTORC1 conducts neuronal cytoskeletal organization and mTORC2, which is located in endosomes and orchestrates lipid metabolism, crucial for genesis of membranes—cellular ‘skin’ and communication platform of all neural cells (Switon et al., 2017). TLRs also induce MAPK cascades, like p38, JNK and ERK which are known to participate in proliferative and differentiative cellular programs in stem cells (Johnson and Lapadat, 2002). MAPK/ERK pathway is IKK-dependant - these pathways could change and dictate daughter cell fate, behavior and metabolism and could be crucial for the timeline of neurodevelopment (Figure 6).

We believe that the hypothesized model introduces the needed complexity and integrative perspective of the mammalian Notch signaling in brain development. Namely, the current model focuses mostly on the signal-receiving cell as the active player, while the role of the signal-sending cell in Notch signaling is not fully understood and is thus overlooked. The current model resembles instruction-giving rather than communication-based decision-making between the cells. We advocate that TLRs untangle the information given by Notch and J/D ligand structure features and provide necessary information and instruction both to signal-sending and signal-receiving cells. Here hypothesized model involving the TLR family in the Notch signaling pathway takes into account two-way intercellular communication in which both cells actively interpret responses in order to efficiently coordinate developmental and homeostatic processes.

## 6 Relevance of the hypothesized Notch/Toll system for the molecular basis of neurodevelopment

Notch signaling is one of the central cellular homeostatic mechanisms able to exert adaptive responses both during development and in mature cells. In this respect, incorporating TLRs and their putative role as fundamental parts of Notch signaling provides a new perspective for neurodevelopment- and neuroimmune-related studies.

It is reasonable to assume that TLRs play a role in two overlapping pathways in the brain—the TLR Notch-dependent (ND) cellular development/homeostasis pathway and the environment-dependent (ED) cellular (immune) response. Differentiation between the two pathways would add to the understanding of molecular aspects of neurodevelopment at the phylogenetic and ontogenic levels. The TLR-ND pathway would be best studied in the course of development to confirm the exact steps of how specific TLR, Notch, and J/D ligands achieve formation of cellular identity and communication during maturation and adulthood when established identity becomes a matter of innate immunity. The TLR-ED pathway is intertwined with ND-mediated cellular crosstalk, homeostatic neural cell-to-cell communication and its environmental influence surveillance by immune cells. In this context, innate immunity proves that TLR proteins are of utter importance for the brain and its homeostasis. In order to prevent cellular miscommunication, microglia, like all innate immunity cells, express the entire TLR repertoire, functioning as an affinity bead that siphons environmental molecular patterns, PAMPs and DAMPs. The immune component of TLR-ED has been widely studied, and some of the phenomena described involve developmental/homeostatic ND pathway. The way the environmental stimuli influence individual cellular TLR-ND and ED pathways will be further discussed.

First, we briefly recapitulate the roles of TLRs and their cellular and time-dependent expression patterns during brain development (Table 1) and propose the following sequence of neurodevelopmental events accompanied by TLRs actions. Let us imagine the developing nervous system and the emerging complexity course depending on the cellular ability to communicate with the environment, more precisely, neighboring cells. TLR scaffold, Jag and Dll ligand type on the signal-sending cell, and available Notch receptor on the signal-receiving cell drive the course of proliferation, differentiation, and specialization, e.g., axon, dendrite, and synapse formation.We suggest that NPC TLR-ND system starts as a simple TLR5/TLR3 answer-reading machine for low-specificity Notch binding events. With increasing number of NPCs, the growing density of TLR3-sensing Notch events may allow the emergence of TLR4, TLR7, TLR8, and TLR9. Hipothetically, this quartet slowly silences proliferation and guides NPC asymmetric division and gradual specialization in progenitor subtypes (radial glia, ventral and basal radial glia, retinal progenitor cells, immediate progenitors, oligodendrocyte progenitor cells). The colonization of the brain is now set off.

Waves of climbing young neurons create layers upon layers, as they travel from densely populated, supposedly high-Notch regions, to unpopulated, presumed low-Notch regions.We anticipate that the juxtacrine input of “older” neurons maintains progenitor cell identity on the way to its destination within growing layers. As development proceeds, documented specific changes of TLRs expression paterns (Bsibsi et al., 2002; Shmueli et al., 2018) suggest that TLR3 could be potentially a progenitor and immature phenotype program switch, while TLR4 and TLR9 a differentiative and mature program switches. When situated, TLR7-driven axonal outgrowth (Ma et al., 2007) creates a foundation for establishing the regional-specific neuronal circuitries and networks. The upcoming gliogenesis builds the cell-type diversity; thus, new TLRs emerge (TLR1 and TLR6) which most likely support the novel identity choice. Once positioned glial cells could shed J/D ligands and convey signal to incoming glial progenitors about area population density, creating regions of cell-diverse clusters. When colonization is complete, maturation can take place. Young neurons start to branch, forming inter-regional connections, and oligodendrocytes start myelination in the presumed TLR-ND fashion, where TLR2 and partners support the ongoing Notch events. Astrocytes and already infiltrated microglia mature and continue supporting decades-long network formation and calibration (Schafer et al., 2012, 2013; Miyamoto et al., 2013; Ikegami et al., 2019). Microglial action acquires the brain’s environmental experience and plastic potential, e.g., network malleability, progenitor number, and potency, providing necessary adaptation mechanisms for survival and growth. TLR proteins react to the differences in environmental stimuli, creating unique cellular makeup, connection variety, cellular mosaicism, and organism-specific circuitry, resulting in various neurophenotypes, behaviors, conditions, and pathologies. To address these varieties, one would need to explore the immune response landscape by tracking TLRs involved in ED and address the influence on the ND counterpart pathway.

Multiple neurodivergent phenotypes and conditions are indicated to stem from environmental challenges during pregnancy and neonatal period of neurodevelopment and involve TLRs. For example, autism spectrum disorder (ASD) (Chung et al., 2021; Yu et al., 2023; Vacharasin et al., 2024), attention-deficit/hyperactivity disorder (ADHD) (Ganna et al., 2018; Kim et al., 2020), schizophrenia and neurodevelopmental disorders (NDDs) are associated with maternal immune activation (MIA)(Baghel et al., 2018; Baines et al., 2020; Talukdar et al., 2021; Anderson et al., 2022). In all mentioned NDDs, specific differences in total brain volume or volume of particular regions compared to controls have been documented by neuroimaging studies (Courchesne et al., 2001; Hyakkoku et al., 2010; Lukito et al., 2020; Li T. et al., 2022). Is it possible that these NDDs are associated with MIA disturbance of TLR-ND pathway? If the TLR challenge coincides with neurogenesis, depending on infectious agent and the spatio-temporal neurodevelopmental stage, there is a firm possibility that diversity in neuronal type and number is introduced. The number of neurons residing in specific brain regions (amygdala, hippocampus, cortex, cerebellum) or even sub-structural clusters, can vary, induce mosaicism, and build the potential for alterations of neuronal networks and connections. As described earlier, bacterial LPS induces neuronal overgrowth (Humann et al., 2016; Mann et al., 2023) and dsRNA silences proliferation (Yaddanapudi et al., 2011). Importantly, the essential role of TLR signaling in neurodevelopment is corroborated by a recent finding of the 16p11.2 deletion syndrome which has phenotypic features of ASD (Chung et al., 2021). One of the affected genes in the 16p11.2 deletion syndrome is coding for ERK1 protein (Pucilowska et al., 2015), a downstream element of the TLR cascade. In other words, ASD neurophenotypes may be exacerbated through inhibition of TLR cascade. Viral and bacterial coinfection effects on the TLR2/4 and TLR3 systems probably participate in molecular events underlying an ASD-described increase and decrease in the volume of particular regions. Persistence and duration of infection could be attributed to the range of ASD phenotypic diversity in individuals.

On the other hand, TLR3 inhibition by viral dsRNA could lower proliferation and neurogenesis capacity which may be associated with decreased number of neurons, atypical cellular density and changed brain volumes in ADHD and schizophrenia. Further, in challenging periods that coincide with neuronal outgrowth and branching involving the TLR7/8/9 system, neurons may shape unusual connections resulting in differential synaptic landscape formation and neural circuitries. The same applies to gliogenesis - if progenitor pool potential is disturbed, the fate of oligodendrocytes and astrocytes can change, and individuals become susceptible to various pathological processes.

When elaborating neuroimmunity in the context of TLRs actions, we should also consider infections caused by the herpes virus family, Epstein–Barr (EBV) and herpes simplex (HSV) virus, and related pathologies, multiple sclerosis (MS) (Hernández-Pedro et al., 2013; Baecher-Allan et al., 2018) and Alzheimer’s disease (AD) (Landreth and Reed-Geaghan, 2009; Cameron and Landreth, 2010). Pathogenesis of MS involves innate and adaptive immunity responses related to demyelination, neuronal loss, and lesion formation (Hernández-Pedro et al., 2013). Potential role of TLRs in MS pathogenesis may be related to viral microRNA. Namely, it has been shown that HSV and EBV regulate immune answer and viral life cycle via viral microRNA-filled extracellular vesicles (Forte and Luftig, 2011; Kim et al., 2017; Wang et al., 2018; Cone et al., 2019). EBV infection in adolescence and later life could affect the pool of brain-residing progenitor cells involving the EBV infected cells release of microRNA, exhibiting immune-and viral cycle-regulating properties (Forte and Luftig, 2011; Wang et al., 2018). It is possible that distinct microRNA, circulating via extracellular vesicles, is taken by OPC and NPC. Once in cells, it could be that microRNAs doublestranded harpins bind TLR3 and dysregulate its activity causing the loss of TLR3 information. This drives progenitor cells to premature differentiation (Yaddanapudi et al., 2011; Bsibsi et al., 2012), apoptosis, and lower plastic ability. The myelinating oligodendrocyte has to be in contact with the neuron, astrocyte, progenitor, and immune cell to successfully fullfill its duty to become a new oligodendrocyte and if not, it is non-invasively removed by microglia (Berghoff et al., 2021). If there is a lack of Notch input due to TLR3 inhibition following a viral infection, the dying OPC/oligodendrocyte identity may be obscured and astrocytes and microglia could mistake it for an invading agent. The resulting innate immunity response might instruct adaptive immunity to attack specific subgroups of oligodendrocytes. We suggest that as adaptive immunity attacks myelin, dying cells produce other TLR ligands (particularly ligands of the TLR2 and TLR4, previously shown to affect regulation of oligodendrocyte life-cycle)(Church et al., 2016; Sanchez-Petidier et al., 2022; Brandt et al., 2023) which continue to disrupt homeostasis, leading to loss of neuronal networks integrity and emergence of MS symptoms as an autoimmune condition. Similarly, sporadic AD is frequent in individuals infected with HSV. The reoccurring infection could be detrimental to the hippocampal progenitor cell pool (Walker et al., 2018) and compromise astrocyte-dependent neuron identity and homeostasis by releasing TLR2 and TLR9 ligands (Lima et al., 2010; Zolini et al., 2014). Interestingly, utilizing a molecular visualization tool, we have observed probable processing of amyloid-precursor protein (APP) similarly to Notch: α-secretase cleavage releases the extracellular domain with two distinct structural motifs. APP E2 domain resembles TLR5 ligand flagellin and functions in *cis*-and *trans*-ligation (Rossjohn et al., 1999; Barnham et al., 2003; Wang and Ha, 2004). The extracellular part of APP has many similarities with the Notch/J/D complex, and APP presence is confirmed in dimers with Notch (Di Chen et al., 2006) (Figure 4B2a). Is it possible that APP is one of the natural Notch and TLR ligands, and its ligation and shedding give information about cellular environment and condition of neurons, resulting with growth and repair? Astrocytes seem to be most affected by miscommunication and may play an essential role in bridging the connection between neurons, oligodendrocytes, and microglia (Krasowska-Zoladek et al., 2007; Guerrero-García, 2020; Werkman et al., 2020; Wang et al., 2021). MS and AD-like pathologies have been previously considered in the context of viral infections, however, taking into account TLR signaling may shed light on events which include cellular response to environmental cues leading to disruption in developing and homeostatic pathways.

Brain tissue damage and injuries are cellular environment-changing events that also affect the microglial TLR system and influence the presumed TLR-ND cellular response. TLR is implicated as an essential player in traumatic injuries, tissue damage, and repair, and its many immunity-related roles in responding to tissue damage have been resolved by studies in genetically modified mouse models with TLR deficiency (Zhang et al., 2011; Ishizuka et al., 2013; Winters et al., 2013; Church et al., 2016; Gorup et al., 2019; Wasko et al., 2019; Polsek et al., 2020). The cellular environment in brain injury becomes saturated with DAMPs, and binding events affect the microglial TLR system, influencing TLR-ND cellular communication (Shi et al., 2013). TLR2 and TLR4 influence post-injury cellular recruitment and axonal tissue regeneration potency (Winters et al., 2013; Gorup et al., 2019). Lack of TLR2 is shown to protect the brain from immune invasion and has a poor prognosis in injury repair and tissue regeneration (Gorup et al., 2019). TLR4 activation/inhibition, on the other hand, has regenerative quality, and aids OPC recruitment and axon remyelination (Schonberg et al., 2007). The basic cellular load of mRNA coding for TLR2-engaged downstream scaffold proteins is differential in TLR2KO mice; an increase in load of IRAK1, IRAK4, IKKβ, and IL-6 and a decrease in IRAK3 has been observed (Winters et al., 2013). Since increased neuronal numbers are a phenotypical trait, we can assume that IRAK1, IRAK4, and IKKβ echo neuronal identity metabolism. At the same time, the observed IL-6 increase could be a constant signal for OPC premature maturation and apoptosis, lowering OPC regenerative capacity.

Finally, TLR and Notch signaling pathways may interfere with modeling and functional effects of hormones during sex-dependent differentiation of the brain. Since the presented data suggests that the Notch-TLR pathway is involved in the formation of cellular identity during neurodevelopment, we could make another intriguing assumption as to whether the building up of individual sex-related behavioral (psychological) patterns depends on the same signaling system, activated in response to myriads of environmental stimuli (Figure 6). Certainly, the resolving in more details the interplay of the environment with central cellular homeostatic and adaptive signaling pathways during neurodevelopment and brain maturation may substantially broaden our understanding of sex-related differences of clinical phenotypes reported for several neurodevelopmental, neurological and psychiatric disorders (ASD, ADHD, schizophrenia, MS, AD) (Krementsov et al., 2014; Carney, 2019; Lins et al., 2019; Hui et al., 2020; Merikangas and Almasy, 2020; Dunstan et al., 2024).

## 7 Conclusion

We proposed a theoretical framework backed up by preliminary modelling of structural and functional assembly of TLRs and Notch which enables highly coordinated actions of both signaling pathways crucial for intercellular communication and cellular adaptation to environment. We point out so far underappreciated roles of TLRs in neurodevelopment and describe striking functional similarities of TLRs and Notch signaling during embryonic and postnatal brain development. Several possible interactions of TLRs and Notch are introduced and evidenced by preliminary analysis of visualized molecular structures which speaks in favor of the hypothesized intriguing cooperation of Notch and specific TLR isoforms in signal-sending and signal-receiving cellular milieu. We expect that a pilot supportive evidence for here presented hypothesis may provide sound basis for revisiting current viewpoints about interplay of TLR and Notch signaling. Future experimental studies, by including some of our proposed approaches, should better delineate the likely fundamental molecular Notch-TLR interactions which in the nervous system may underlie formation of cellular identity, cytoarchitecture, neural circuitries and diverse neurobehavioral phenotypes.

## Data Availability

The original contributions presented in the study are included in the article/supplementary material, further inquiries can be directed to the corresponding authors.

## Footnotes

^1^ https://www.rcsb.org/

^2^ https://www.ebi.ac.uk/msd-srv/ssm/

^3^ https://www.cgl.ucsf.edu/chimera/
